# Asbestos exposure as an additional risk factor for small duct intrahepatic cholangiocarcinoma: a pilot study

**DOI:** 10.1038/s41598-023-27791-1

**Published:** 2023-02-13

**Authors:** Francesco Vasuri, Marzia Deserti, Angelo G. Corradini, Simona Tavolari, Valeria Relli, Andrea Palloni, Giorgio Frega, Stefania Curti, Stefano Mattioli, Matteo Cescon, Antonia D’Errico, Giovanni Brandi

**Affiliations:** 1 Pathology Unit, IRCCS Azienda Ospedaliero-Universitaria di Bologna, Via Albertoni 15, Bologna, Italy; 2 Department of Specialty, Diagnostic and Experimental Medicine, University of Bologna, Bologna, Italy; 3 Oncology Unit, IRCCS Azienda Ospedaliero-Universitaria di Bologna, Via Albertoni 15, Bologna, Italy; 4 Department of Medical and Surgical Sciences, University of Bologna, Bologna, Italy; 5 Department of Environmental and Prevention Sciences, University of Ferrara, Ferrara, Italy; 6 General and Transplant Surgery Unit, IRCCS Azienda Ospedaliero-Universitaria di Bologna, Via Albertoni 15, Bologna, Italy

**Keywords:** Bile duct cancer, Cancer epidemiology, Histology

## Abstract

Intrahepatic cholangiocarcinoma (iCCA) is a rare malignancy, recently classified in small duct and large duct morphological subtypes. Growing evidence suggests asbestos as a putative risk factor for iCCA, albeit no correlation between asbestos and iCCA morphology has been investigated so far. The aim of the present study was to assess the relationship between asbestos exposure and iCCA morphological subtype. Forty patients with surgically removed iCCA were prospectively enrolled: asbestos exposure was assessed according to the Italian National Mesothelioma Register questionnaire. From the surgical iCCA specimens the main histopathological variables were collected, including the small duct (sd-iCCA, 32 patients) and large duct subtypes (ld-iCCA, 8 patients). Five sd-iCCA cases had a definite/probable occupational exposure to asbestos, while no cases of ld-iCCA were classified as being occupationally exposed (definite/probable). Other kind of asbestos exposure (i.e. possible occupational, familial, environmental) were recorded in 16 sd-iCCA and 3 ld-iCCA. Cases with unlikely exposure to asbestos were 11 sd-iCCA (35.5%) and 5 ld-iCCA (62.5%). In conclusion, these findings seem to indicate that sd-iCCA might be more frequently associated to asbestos exposure rather than ld-iCCA, suggesting that asbestos fibres might represent a *parenchymal*, rather than a ductal risk factor for iCCA. This pilot study must be confirmed by further case–control studies or large independent cohorts.

## Introduction

Intrahepatic cholangiocarcinoma (iCCA) represents the second primary liver cancer (PLC) in incidence, accounting for 10–15% of PLCs^1^. Albeit in Europe and USA iCCA is still considered a rare malignancy, its incidence is increasing, with a large variability worldwide, which reflects the underlying different risk factors: the high incidence observed in Eastern Countries has been related to the endemic diffusion of liver fluke infection, hepatolithiasis and chronic biliary^2^; conversely, in the Western Countries, up to 40% of iCCA patients have not recognizable risk factors^3–5^ and recently, in Japan, it has been considered as an occupational disease among printers due to solvent exposure^6^. Interestingly, a link between aetiology and iCCA histotype has been utterly demonstrated: according to the most recent WHO classification, iCCAs of the small duct subtype (sd-iCCA) are related to non-biliary cirrhosis and chronic hepatitis (i.e., the same intrahepatic risk factors of hepatocellular carcinomas), whereas iCCAs of the large duct subtype (ld-iCCA) are related to biliary chronic diseases, cholangitis, hepatolithiasis and liver fluke infections (i.e., the same biliary risk factors of extrahepatic and hilar cholangiocarcinomas)^1^.

**Figure 1:**
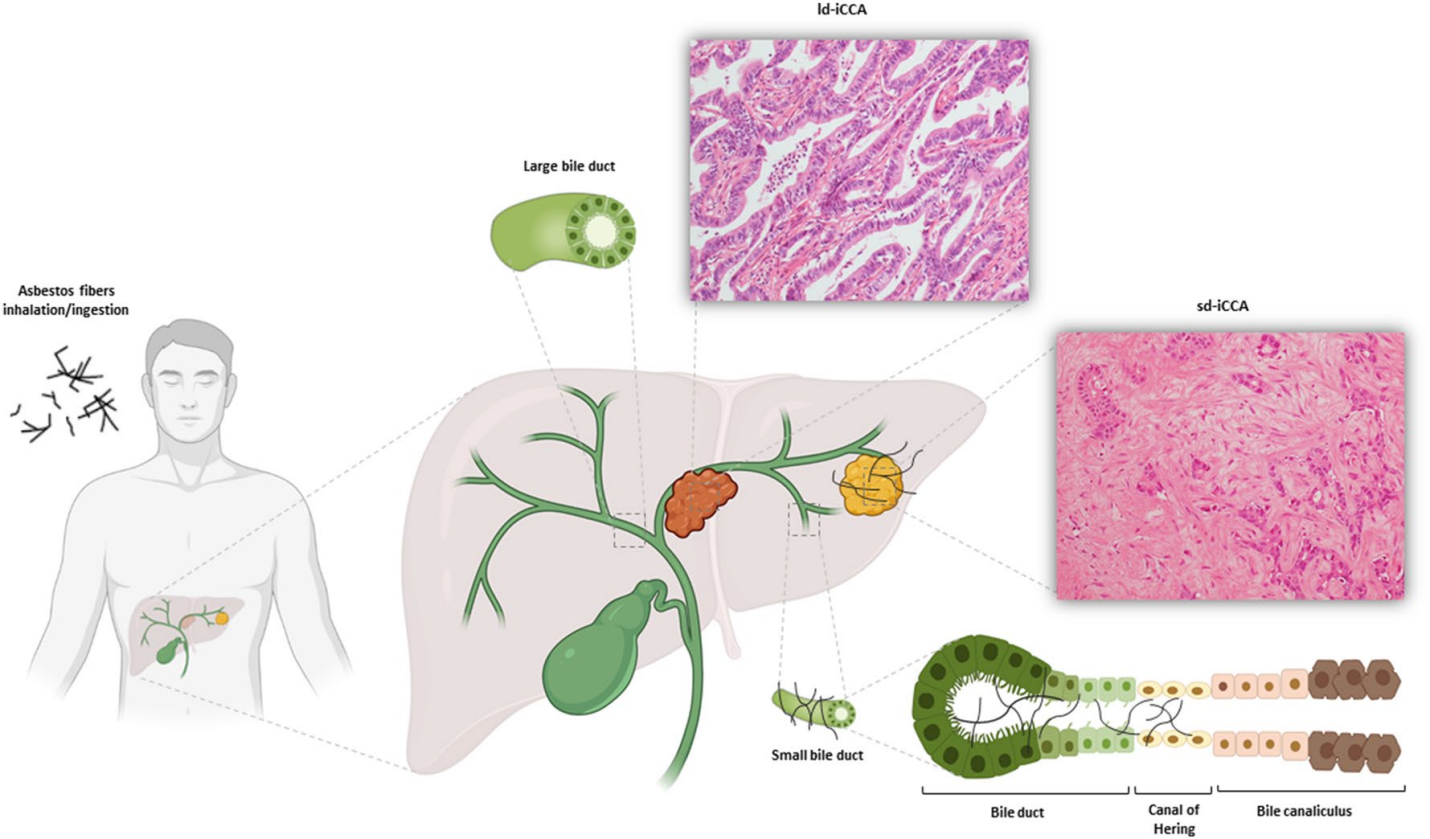
Schematic picture of the relationship between asbestos exposure and small duct iCCA. Highly-penetrant asbestos fibers—deriving from inhalation or ingestion reach the deepest liver parenchyma through the blood stream, up to the Hering’s channels and the terminal bile ductules, giving rise to iCCA with small duct morphology. Large ducts are not included in this pathway.

Recent studies suggested an increased risk of CCA in patients occupationally exposed to asbestos fibers. Farioli et al. observed an increased risk of CCA in a case–control study nested in the Nordic Occupational Cancer (NOCCA) cohort in four Nordic countries^7^. The association between asbestos and cholangiocarcinoma was strongest for iCCA than extrahepatic cholangiocarcinoma (eCCA). This evidence confirmed the results of a previous Italian case–control study^8^, indicating a fourfold risk of developing iCCA (higher than the risk of developing extrahepatic CCA) in patients with previous occupational exposure to asbestos. Indeed, some studies carried out in an Italian area with high asbestos exposure (Casale Monferrato, Piedmont) have demonstrated the presence of fibers within the patients’ biliary tree and gallbladder^9,10^ and, more interestingly, in the tumor tissue of iCCA patients^11^. How asbestos inhaled or ingested fibers could reach the liver parenchyma is not clear. The most reasonable hypothesis is that the fibers might be able to reach the bloodstream and then all organs through the pulmonary and the portal lymphatic system (Fig. 1)^11,12^. In the liver, asbestos fibers may induce a chronic inflammation, which unpairs the balance between cell proliferation and apoptosis^13,14^. Moreover, the thinner asbestos fibers might remain trapped in the smaller bile ducts more easily that in the largest ones; this could explain the difference in correlation between asbestos and iCCA *versus* eCCA^7,8^, while no evidence still exists on asbestos as a risk factor for hepatocellular carcinoma. Just a report of five cases has been published on this matter^15^.

Despite the suggestion of an increased risk of iCCA in patients exposed to asbestos, no correlation between iCCA morphology (i.e. sd-iCCA *versus* ld-iCCA) and asbestos exposure has been investigated so far. The aim of the present study was to assess the relationship between asbestos exposure and iCCA morphological subtypes.

## Results

### Patients and risk factors

Forty patients were enrolled, 18 (45%) males and 22 (55%) females, all submitted to liver resection for histologically-proven iCCA, with mean age at surgery of 63.9 ± 12.1 years (range 36–85 years). All clinical characteristics are listed in Table 1.

**Table 1:** Main clinical characteristics of the 40 enrolled patients.

Patients’ characteristics	N (%)
Tumor stage	
Stage I	9 (22.5%)
Stage II	9 (22.5%)
Stage III	14 (35.0%)
Stage IV	2 (5.0%)
n.d.	6 (15.0%)
Surgical margin status	
R0	21 (52.5%)
R1	13 (32.5%)
n.d.	6 (15.0%)
Lymph node involvement	
N+	9 (22.5%)
N0	24 (60.0%)
n.d.	7 (17.5%)
Systemic treatment	
Yes	30
No	10
Serum CA 19.9 at diagnosis	
> 37 U/ml	17 (42.5%)
< 37 U/ml	20 (50.0%)
n.d.	3 (7.5%)
Hepatitis profiles	
HCV	3 (7.5%)
HBV	2 (5.0%)
Autoimmune	2 (5.0%)
NO hepatitis/other	33 (82.5%)
History of alcohol consumption	
Yes	8 (20.0%)
No	29 (72.5%)
n.d.	3 (7.5%)
Smoke abuse	
Yes	23
No	17
Diabetes mellitus	
Yes	4 (10.0%)
No	33 (82.5%)
n.d.	3 (7.5%)

*n.d.* not determined.

According to the ReNaM guidelines, asbestos exposure was classified as definite/probable occupational exposure in 5 (12.5%) patients, possible occupational exposure in 6 (15.0%) patients, environmental exposure in 6 (15.0%) patients, familial exposure in 8 (20.0%) patients, and unlikely in 16 (40.00%) patients. The mean duration of exposure to asbestos was 34.3 ± 15.2 years (range 10–66 years). These long (or very long) asbestos exposures were characterised by low (or very low) concentration of asbestos fibres.

Twenty-two (55.0%) patients had hepatobiliary risk factors: hepatolithiasis/choledocholithiasis in 8, alcoholic chronic hepatitis in 9, HBV chronic hepatitis in 2, HCV chronic hepatitis in 3; in addition, steatosis was associated in 11 cases.

Combining the different risk factors of the enrolled patients, 9 (22.5%) had no known risk factors, 10 (25.0%) had hepatobiliary risk factors (3 hepatolithiasis, 4 alcoholic, 1 HBV and 2 HCV), 12 (30.0%) had asbestos exposure plus hepatobiliary risk factors (5 hepatolithiasis, 5 alcoholic, 1 HBV and 1 HCV), and 8 (20.0%) had asbestos exposure but no hepatobiliary risk factors. No differences were recorded in the distribution of the other risk factors between exposed and not exposed patients (*data not shown*).

### Histopathological features of iCCAs with and without asbestos exposure

No significant differences were recorded between the asbestos exposure groups with respect to age (*p* = 0.20, Kruskal–Wallis test), sex (*p* = 0.44, Pearson’s chi-squared test) and occurrence of risk factors other than asbestos exposure (*p* = 0.987, Pearson’s chi-squared test, see above).

At histopathological analysis, 32 (80.0%) tumors were sd-iCCA subtype, 8 (20.0%) were ld-iCCA. MVI was present in 17 (42.5%). WHO tumor grade was: grade 1 in 3 (7.5%), grade 2 in 22 (55.0%), and grade 3 in 14 (35.0%). All iCCA were of the tubular histotype, excluding 3 prevalently solid cases (2 sd-iCCA and 1 ld-iCCA) and 1 mucinous ld-iCCA.

The distribution of iCCA subtypes according to asbestos exposure is shown in Table 2. Of note:

- Definite/probable occupational asbestos exposure was recorded in 5 cases, all sd-iCCA, while no cases of ld-iCCA were recorded among those patients classified in this category of exposure.
- cases with possible occupational exposure were 5 sd-iCCA and 1 ld-iCCA. Taken altogether, cases exposed to asbestos (including occupational, environmental and familial exposure) were 21 (65.6%) sd-iCCA patients and 3 (37.5%) ld-iCCA patients.
- Cases with unlikely exposure to asbestos were 11 (34.4%) sd-iCCA and 5 (62.5%) ld-iCCA (exposed vs not exposed: *p* = 0.229 at Fisher’s exact test; OR: 3.2, 95% CI [0.6–15.9]).
- The OR (adjusted for age, gender, alcohol drinking, smoking status and HBV/HCV chronic hepatitis) for being exposed to asbestos was 3.4 (95% CI 0.6–93.7) for sd-iCCA patients, considering ld-iCCA patients as reference.

**Table 2:** Distribution of the small-duct (sd) and large-duct (ld) subtypes of iCCA among enrolled patients according to asbestos exposure categories based on the ReNaM guidelines.

Asbestos exposure	Subtype	
	sd-iCCA	ld-iCCA
Unlikely	11 (34.4%)	5 (62.5%)
Environmental exposure	6 (18.8%)	2 (25.0%)
Familial exposure	5 (15.6%)	0 (0.0%)
Occupational (possible) exposure	5 (15.6%)	1 (12.5%)
Occupational (definite/probable) exposure	5 (15.6%)	0 (0.0%)
Total	32 (100.0%)	8 (100.0%)

No differences among the exposure groups were found as far as the other histopathological variables are concerned (*data not shown*).

## Discussion

Our results showed that iCCAs of the small duct subtype (sd-iCCA) might be more likely to be exposed to asbestos than iCCAs of the large duct subtype (ld-iCCA) (65.6% vs 37.5%). To be noted that in a published case–control study the proportion of control subjects selected among the general population with an occupational exposure to asbestos was 11.0%^8^; however, in the present study 31.2% of sd-iCCa patients had an occupational exposure to asbestos, whereas only 1 case (12.5%) was recorded among large duct subtype (ld-iCCA). This is noteworthy, since sd-iCCA has been correlated rather with the main *hepatocellular* (or *parenchymal*) risk factors (like viral hepatitis), than *biliary* (i.e. ductal) risk factors, like cholelithiasis^16,17^, which lead to ld-iCCA as well as eCCA. Our results seem to suggest that a long duration of asbestos exposure–alone or in combination with other established risk factors-may represent an additional *parenchymal* risk factor for sd-iCCA: this is fully in agreement with the previous results by Farioli et al. and Brandi et al., which found an increased risk of iCCA in patients exposed to asbestos fibers^7,8^. Recent models suggested that highly-penetrant thin asbestos fibers –deriving from inhalation (general circulation) and/or digestive tract (portal circulation)– can precipitate in the deepest liver parenchyma, up to the Hering’s channels and the terminal bile ductules, and may induce oncogenesis by different mechanisms (Fig. 1)^18^. One of the most intriguing hypotheses is that the fibers have an oncogenic effect directly on the hepatic stem cells, which reside in the Hering’s channels^19^. The direct damage on stem cell niche might also explain why sd-iCC have no known morphological precursors, unlike ld-iCC and eCC, which can be found together with intraepithelial neoplasia of the biliary tract (BilIN)^1^. However, indirect damage plays a role as well: thin fibers > 20 µm in length may promote a prolonged chronic inflammation, since macrophages are not able to phagocyte them completely, maintaining a pro-inflammatory environment^20^. In this scenario, also Kupffer cells are likely to play a role in fibers deposition and chronic inflammation maintenance^18^. The major limitation of this study is the small sample size that decreases the statistical power of the study. This limits the interpretation of the results (including the adjusted analysis) precluding any causal conclusions. In addition to that, there is a variety of risk factors for iCC that are not related to asbestos exposure, as already reported^3^. Moreover, recall bias due to the use of a questionnaire to evaluate exposure data must be considered. In particular, considering the small sample size, recall bias could have resulted in an underestimation of asbestos exposure among ld-iCCA patients. A selection bias could be considered as well; however, our monocentric study was located in a hospital that is a major catchment centre for bile ducts neoplasms and patients were enrolled prospectively. Consequently, it is unlikely that selection bias might have contributed to the difference between asbestos exposure proportion in sd-iCCA and ld-iCCA.

Nevertheless, this study reinforces the putative association between asbestos exposure and iCCA^7,8^ suggesting for the first time that the exposure to asbestos might be more likely to be related to the small-duct histological subtype of iCCA than to the large-duct subtype. This could be of paramount importance, considering that the environmental exposure to asbestos tends to be underestimated or unknown. Further case–control studies or large independent prospective cohort studies are needed in order to validate this pilot study, considering other established risk factors for iCCA.

## Methods

The study group included patients from EtherBil study (registration code NCT02184871 on ClinicalTrials.gov): this is a monocentric prospective study which received approval from the local Ethical Committee (Comitato Etico Area Vasta Emilia Centro, CE-AVEC) with identification code EM221-2019_21/2014/U/Tess/AOUBo del 20/03/2019. Patients signed the informed consent for the study at the moment of surgery. Those who underwent a surgically-removed iCCA (with availability of tumoral tissue) were prospectively enrolled. A structured standardized questionnaire was administered by trained interviewers to cases or their next of kin; it includes the collection of occupational and residential history and information on lifestyle habits as well as clinical data.

The questionnaire includes three separate sections:

- *Section 1*, general information, including personal data, lifestyle and habits.
- *Section 2*, corresponding to the questionnaire of the Italian National Mesothelioma Register (Registro Nazionale dei Mesoteliomi, ReNaM): it collects information on work history, family anamnesis, environmental and domestic exposure, and non-occupational exposure to asbestos^21,22^.
- *Section 3*, clinical history, including hepatic and not hepatic pre-existent diseases and established risk factors.

The assessment of asbestos exposure was carried out by an occupational physician (SM) according to the ReNaM guidelines^23^. The lifetime asbestos exposure was classified as: definite/probable occupational exposure, possible occupational exposure, environmental exposure, familial exposure, or unlikely asbestos exposure.

**Figure 2:**
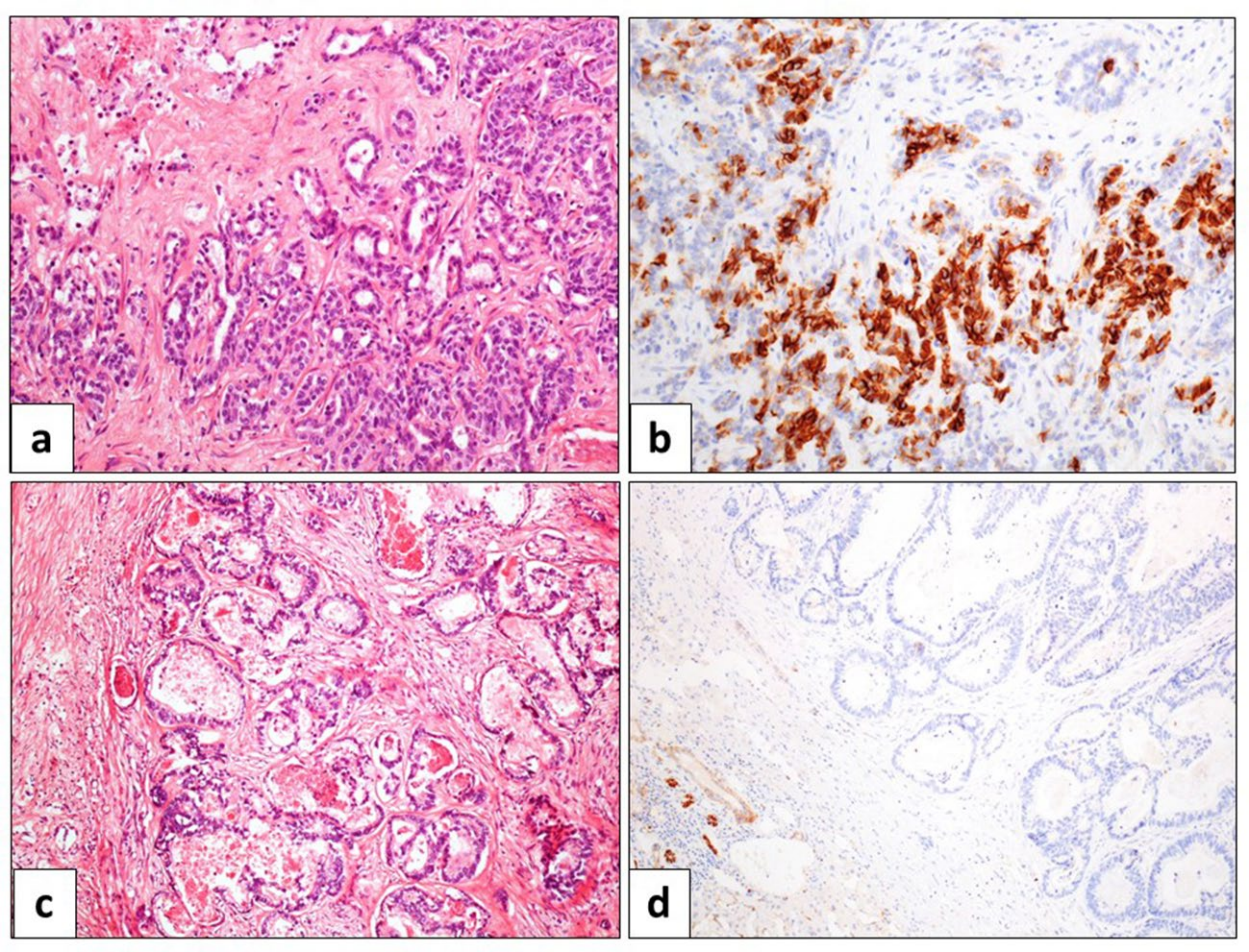
Pathological features of the two iCCA subtypes. (**a)** Haematoxylin–Eosin stain) iCCAs of the small-duct subtype show small and regular ducts and tubules, lined by a cuboidal epithelium with generally few atypia, and surrounded by a dense and desmoplastic stroma. (**b**) Immunohistochemistry for CD56 is generally positive, as suggested by the most recent guidelines. (**c)** Haematoxylin–Eosin stain) iCCAs of the large-duct subtype show large and branching ducts, lined by variable cells, from cuboidal to columnar, with variable amount of mucinous component. (**d**) Immunohistochemistry for CD56 is typically negative. Magnification 20x.

Histological analysis was performed from routinely formalin-fixed paraffin embedded material from all surgical specimens. At Haematoxylin–Eosin tumor grade, the occurrence of microvascular invasion (MVI), and the histological histotype were recorded, including the small duct and large duct subtypes (sd-iCCA and ld-iCCA) according to the most recent WHO guidelines^1^. In detail, sd-iCCA is characterized by a ductal or tubular pattern, with a variable amount of desmoplastic stroma; these neoplastic ducts are lined by cuboidal or columnar cells with variable pleomorphism, inconspicuous nucleoli and scarce cytoplasm. ld-iCCA is generally very similar to eCCA in morphology, with larger and branching neoplastic ducts within a desmoplastic reaction, secretive cell with occasional mucin deposition, and frequently lymphovascular and/or perineural invasion (Fig. 2).

Immunohistochemistry for Keratin 7 (K7, rabbit monoclonal, clone Sp52, CC1S, prediluted; Roche), Keratin 19 (K19, mouse monoclonal, clone A53-B/A2.26, prediluted; Cell-Marque), and CD56 (mouse monoclonal, clone 123C3.D5, prediluted; Roche) was applied in course of routine diagnosis when required, by means of the automated immunostainer Benchmark® ultra (Ventana Medical Systems, Inc, Roche group, Tucson AZ, USA). In particular, K7 and K19 were used when a confirmation of the iCCA diagnosis was required; CD56 was used to confirm the sd-iCCA subtype when required^1,24^.

All methods used to conduct the study were performed in accordance with relevant guidelines and regulations.

### Statistical analysis

All analyses were carried out using STATA 16 (Stata corporation, Texas, TX, USA). Data are reported as means ± standard deviations, ranges and frequencies. Pearson’s chi-squared test, Fisher’s exact test and Kruskal–Wallis tests were applied, as appropriate. In addition, to evaluate the risk of being exposed to asbestos for sd-iCCA patients, a multivariable unconditional logistic regression model was fitted controlling for age (coded in three categories: ≤ 55, 56–65, ≥ 66 year), gender, alcohol consumption (drinkers/non-drinkers), smoking status (ever/never smokers), HBV/HCV chronic hepatitis (yes/no). Patients with ld-iCCA were taken as the reference category. We estimated OR and 95% confidence interval (95%CI) according to Breslow and Day^25^. An α error = 0.05 was accepted.

## Supplementary Information

Supplementary Information.

## Data Availability

The dataset used and analyzed during the current study are available from the corresponding author on reasonable request.
