# Supplementary material for: Asbestos exposure as an additional risk factor for small duct intrahepatic cholangiocarcinoma: a pilot study
Source: Sci Rep. 2023 Feb 13;13:2580. doi: 10.1038/s41598-023-27791-1 (PMC9925780; doi:10.1038/s41598-023-27791-1)
Supplement: Supplementary file 1 — Supplementary Information. [file 41598_2023_27791_MOESM1_ESM.pdf]

ISPESL

Italian National Institute for Occupational Safety and Prevention

Occupational Medicine Department

Epidemiology Unit

Mesothelioma National Register (Article 36, D.Lgs.n°277/91 – Dpcm308/02)

GUIDELINES FOR THE IDENTIFICATION AND DEFINITION OF MALIGNANT MESOTHELIOMA CASES AND THE TRANSMISSION TO ISPESL BY REGIONAL OPERATING CENTRES – Translation from the Italian version by Dr. Claudia Branchi

PREFACE

Prime Minister's Decree no.308 dated 10 December, 2002 was published in the Official Journal No.31 dated 7 February, 2003.

The decree establishes the "Regulations for the definition of the model and the procedures for keeping the register of asbestos-related mesothelioma cases" set out by the Legislative Decree no. 277/1991 which in the article 36 – – *"Cancer Register"– provides for "the setting up of a register of recognized cases of asbestosis and asbestos-related mesothelioma at the Italian National Institute for Occupational Safety and Prevention (ISPESL)".*

The Decree No. 308 enacts the conclusion of the legislative proceding that entrusted ISPESL with the setting up of the National Register of Mesotelioma cases (ReNaM)

The need to establish a nation-wide system for malignant mesothelioma monitoring system arises from the massive utilization of asbestos which took place in Italy in numerous economic activity sectors up to the 1980s and from the severe effects for public health. The asbestos ban introduced by the Legislative Decree no. 277/1992 has not completely deployed its beneficial effects on public health yet, considering the long latency period of mesothelioma and the widespread presence of asbestos not only in the economic activity sectors traditionally asbestos associated but also in the environment.

Incidence and temporal trends place this pathology among those of greatest interest in terms of primary prevention.

The "Guidelines for the Identification and Definition of malignant mesothelioma cases and Transmission of Information to ISPESL by the Regional Operating Centres" published in 1996 have proved to be a precious aid to the experts. During these years, notwithstanding the lack of the regulation implementing the D.Lgs. no.277/1991, a series of regional experiences pursued in conjunction with the ISPESL, have pursued an important scientific activity concerning the epidemiological surveillance of mesothelioma.

Five Italian regions initially established and provided data to ReNaM (Piemonte, Liguria, Emilia-Romagna, Toscana and Puglia. During the past three years 6 further regions (Lombardia, Veneto, Sicilia, Marche, Basilicata and Campania) joined the initial group. Currently ReNaM is an epidemiological surveillance system operating over the 60% of the national territory, with more than 42 million of residents and involving regions that in the period 1988-1977 recorded a number of pleural cancer deaths covering the 80% of national cases.

The development of modern diagnostic techniques and the increasing knowledge concerning new possible exposure opportunities required the revision and the updating of the guidelines.

Important modifications have been introduced, with respect to the previous edition, concerning the attribution of criteria to the various levels of diagnostic certainty. In addition the questionnaire has been further implemented for the collection of data concerning the working and residential history of the subjects affected by the disease.

We hope that these guidelines may continue to be a useful aid to the experts that are involved in the epidemiological surveillance of malignant mesothelioma in Italy and that they may facilitate the extension of the epidemiological surveillance activity thorough the national territory through the setting up of the Regional Operating Centres.

Massimo Nesti

ISPESL - Occupational Medicine Department Epidemiology Unit

National Institute for Occupational Safety and Prevention (ISPESL)

Occupational Medicine Department
Epidemiology Unit

INDEX

Preface

Legislative framework
Objectives of National Mesothelioma Register (ReNaM) Basic information contained in the Register
Statistics survey Unit
System architecture and Regional Operating Centres Data collection, implementation and registration Diagnostic definition of cases
Asbestos exposure definition Technical Committee
The "National Institute for Occupational Safety and Prevention" (ISPESL)

Chapter 1 - Organization of an information network for the detection of cases

1.1 Operating Centres and Data sources
1.2 Operative pattern for cases investigation and reporting.

Chapter 2. – Diagnosis definition

2.1 Definition criteria of cases in ReNaM
2.2 Priority in the exposure definition
2.3 Definition criteria of incidence date of cases in ReNaM
Supplementary Table 2.1 Summary of classification criteria of cases reported in ReNaM

Chapter 3. – The questionnaire

3.1 Description of contents
3.2 Instruction for the compilation of the questionnaire
3.3 The questionnaire
3.4 Exposure definition criteria

Chapter 4. – The Interview and the Interviewers

4.1 Introduction

4.2 Preparing the interview

4.3 Interview guidelines

4.4 Interview phases

Chapter 5. Asbestos-related Mesothelioma National Register

5.1 Notification of Malignant Mesothelioma cases to the National Register

5.2 ReNaM software (relase 2.0) for automatic recording and forwarding of data: features and operating instruction

5.3 ReNaM activities and initiatives in collaboration with Regional Operative Center

Appendix

1) DPCM n° 308/2002

2) Operating guide of ReNaM software (release. 2.0)

INTRODUCTION

Legislative context

Malignant mesothelioma, is a pathology with a high level of mortality and short survival associated to asbestos exposure.

Notwithstanding the publication of the Legislative Decree no. 257 dated 27 march 1992 concerning "the regulations pertaining to the ban on the use of asbestos", it is reasonable to envisage that, considering the large quantities of asbestos used and the long latency period of malignant mesothelioma, the beneficial effects of the Decree 257/92 shall be perceived for still a long time to come.

Five European directives issued between 1980 and 1988 were implemented in Italy on 15 August 1991 with the promulgation of Legislative Decree 277 concerning the protection of workers against risks arising from exposure to physical, chemical and biological agents in the workplace, with special attention to lead, asbestos and noise.

Following the implementation of Article 17 of Community Directive No. 83/477 setting out "for Member States the obligation to keep a register of recognized cases of asbestosis and mesothelioma", on 15 August 1991 Legislative Decree 277 was issued in Italy which in

Article 36 – “Cancer Register”– provides for “the setting up of a register of recognized cases of asbestosis and asbestos-related mesothelioma at the Italian National Institute for Occupational Safety and Prevention (ISPESL)”. The “Rules for the definition of the model and the procedures for keeping the register...” were set out by Decree of the President of the Council of Ministers no. 308 dated 10 December, 2002 published in Official Journal No. 31 dated 7 February, 2003

ISPESL in conjunction with regional experts having a considerable experience in this subject, has drown up in 1996 operating Guidelines for the definition, collection and transmission of malignant mesothelioma cases sent by the CORS to ISPESL .

This is a revised and updated version of the guidelines.

Objectives of ReNaM-Registro Nazionale dei Mesoteliomi (National Mesothelioma Register).

ReNaM objectives are:

a) to establish mesothelioma incidence in Italy;

b) to acquire information relating to previous asbestos exposure of registered cases;

c) to contribute to the assessment of the effects of industrial use of asbestos and to the identification of sources of pollution;

d) to promote research projects for the assessment of the association of mesothelioma cases and asbestos exposure.

Basic information contained in the Register

The following basic information must be reported in the National Register:

- personal data
- cancer site
- disease diagnosis and identification date
- professional history
- data concerning cohabitants’ exposure to asbestos
- data concerning residential background and hobbies
- data sources.

Statistics collection units

The statistics collection units, conceived as the primary input of the procedure, are all cases of malignant mesothelioma of the pleura, peritoneum, pericardium and tunica vaginalis of the testicle, including suspected cases.

System architecture and Regional Operating Centres

The flow of information linked to the acquisition of mesothelioma cases and the definition of exposure proceeds along a series of predetermined information channels through which it is possible to raise the informational value of elementary data obtained in the initial phase of the procedure. The system architecture provides for the role of *Regional Operating Centres (CORs)* as a sort of link between the reporters of cases and the

National Institute of Occupational Safety and Prevention.

These Units act as a hub for information coming from different archives and ensure the thoroughness and quality of this information. In five Italian Regions (Piedmont, Liguria, Emilia-Romagna, Tuscany, Puglia) the Regional Health Departments have already identified the CORs responsible for reporting mesothelioma cases and verifying past exposure to asbestos. CORs are established within *Regional Epidemiological Observatories* or other epidemiological Services, *Local Mesothelioma Archives, Population-based Cancer registries* or other similar structures already operational.

CORs activities:

Acquisition, processing and filing of information (phases 1, 2, 3)

All sources with special interest - public hospitals and private clinics, university departments, pathologic anatomy services and institutes, local health authorities, national higher institutes, etc. - send to the Regional CORs the available documentation pertaining to each case (including suspected cases) of malignant mesothelioma. Parallel to this passive activity, CORs conduct active research of cases from potentially suitable sources through the direct consultation of archives.

Diagnostic definition of cases

The relative rarity of malignant mesothelioma and the complexity of histological aspects make the diagnosis of such cases rather difficult. For this reason reference *Diagnostic*

protocols have been drafted to permit, whenever possible, the standardised diagnosis of mesothelioma. A standard interpretative grid allows a breakdown of cases into classes or groups depending on the degree of diagnostic approximation. The application of a minimum admissibility criterion makes it possible to extrapolate confirmed cases in order to move on to the next phase, exposure definition to asbestos. At the same time, consistency controls have been fixed, i.e. criteria and procedures aimed at assessing diagnostic uniformity through a critical revision of the diagnoses received or recorded.

Definition of asbestos exposure

The analysis of the patient's professional history, together with information on lifestyle and residential history, is conducted in accordance with a specific protocol including the compilation of a standard questionnaire, for which the interviewer has been trained in advance. The interview is directly with the patient (direct interview) or, having verified his unavailability, with a relative (indirect interview).

The standard classification including different levels enables to asses, according to homogeneous criteria, the presence (or absence) of asbestos exposure, and to assign each case to various levels of probability in relation to asbestos exposure.

If the information acquired through the questionnaire does not permit a precise and unmistakable judgement about environmental or occupational exposure, a further phase of study is conducted. The opinion on the thoroughness and reliability of acquired information is verified through a process of revision and critical appraisal of the compiled questionnaire.

CORs collaborate with local health and public hygiene structures and with occupational prevention, hygiene and safety services for the collection of data on the occupational and residential exposure of identified cases.

The technical Committee

A *Technical Committee* has been set up within the *National Institute of Occupational Safety and Prevention (ISPESL)*, made up of pathologists, epidemiologists, clinicians, hygienists and representatives of CORs, trade unions and employers associations as well as of other public institutions.

This *Committee* has the tasks of:

- establishing and updating standards for the acquisition of information *(procedures, information flows)*;
- determining processes for verifying the quality of diagnoses and working histories;
- drawing up plans for periodical checks on information procedures;
- fixing methods for the processing of information in accordance with the objectives to be achieved;
- defining the ways in which information is disseminated and used for further researches in compliance with confidentiality constraints.

In carrying out these tasks, an ongoing debate is conducted within the *Committee* on these issues, with the aim of making the best use of what has been learnt over time about the pathology and its correlation with asbestos exposure. Expiring the first three-year term of office, the Technical Committee is waiting to be reappointed.

National Institute of Occupational Safety and Prevention (ISPESL).

The Institute has set up a procedure for the acquisition, processing and filing of information received. On the basis of criteria fixed by the Technical Committee, ISPESL elaborates and regularly updates *reference standards* for the detection and registration of cases. On this point, reference Guidelines have been drawn up for the implementation of the information network and for the diagnostic and anamnestic definition of cases.

The Institute acts as a sorting office for information among CORs in cases of health migrations (i.e. persons addressing to health centres operating in regions different from the residence) and, moreover, provides CORs with useful elements for reconstructing the working history of subjects through an agreement with social security institutes. ISPESL also intends to encourage the work performed by CORs, promoting epidemiological studies on quantitative assessments of risks for exposed populations (both working and non-working), the identification of worker categories exposed to asbestos for whom exposure has so far gone unrecognised, and the identification of other agents that might be the cause of mesothelioma.

CRITERIA FOR DIAGNOSTIC DEFINITION

All cases of malignant mesothelioma, even suspected are included and therefore recorded in the National Mesothelioma Register. The case classification provides for 5 groups and

several subgroups of decreasing levels of diagnostic certainty, in relation to the procedure and diagnostic certainty achieved:

1. malignant mesothelioma that is CERTAIN (with 3 subgroups)
2. PROBABLE malignant mesothelioma (with 2 subgroups)
3. POSSIBLE malignant mesothelioma (with 2 subgroups)
4. malignant mesothelioma TO BE DEFINED (with 3 subgroups)
5. NOT malignant mesothelioma

Supplementary Table 1 briefly shows the diagnostic examinations required and their certainty for including the cases in the various groups and subgroups.

1 CERTAIN MALIGNANT MESOTHELIOMA

1.1 The case must be characterised by all the following conditions:

- Microscopic examination on material (histological or cytological with centrifugation of the sediment) enclosed in paraffin, with characteristic morphological pattern. Tissues removed during a post-mortem examination are also included;
- Immunohistochemistry with characteristic immunophenotypical pattern;
- Diagnostic imaging (confirmation of primary pleural or peritoneal neoplastic lesion and exclusion of alternative pathology) or else diagnosis of discharge of mesothelioma or similar assessment made by a clinician.

1.2 The case must be characterised by all the following conditions:

- Microscopic examination on histological material enclosed in paraffin, with characteristic morphological pattern. Tissues removed during a post-mortem examination are also included;
- Immunohistochemistry not carried out or pattern not defined;
- diagnostic imaging (confirmation of primary pleural or peritoneal neoplastic lesion and exclusion of alternative pathology) or else diagnosis of discharge of mesothelioma or similar assessment made by a clinician.

1.3 The case must be characterised by all the following conditions:

- Microscopic examination on histological material enclosed in paraffin, with characteristic

morphological pattern. Tissues removed during a post-mortem examination are also included;

- Immunohistochemistry not carried out or pattern not defined;
- Absence of clinical and radiological data available to the Regional Operative Centre (COR) to assess the legitimacy of clinical diagnosis.

2 PROBABLE MALIGNANT MESOTHELIOMA

2.1 The case must be characterised by all the following conditions:

- Histological or cytological examination with enclosure in paraffin carried out, even during an post-mortem examination, but which did not give a result indicating mesothelioma in a clear and reliable way (doubtful case);
- diagnostic imaging (confirmation of primary pleural or peritoneal neoplastic lesion and exclusion of alternative pathology) or else diagnosis of discharge of mesothelioma or similar assessment made by a clinician.

2.2 The case must be characterised by all the following conditions:

- Cytological examination not enclosed in paraffin, carried out even during a post-mortem examination, with characteristic pattern and report expressed in terms clearly indicative of mesothelioma (and not of simple malignant neoplasia);
- Diagnostic imaging (confirmation of primary pleural or peritoneal neoplastic lesion and exclusion of alternative pathology) or else diagnosis of discharge of mesothelioma or similar assessment made by a clinician.

3 POSSIBLE MALIGNANT MESOTHELIOMA

3.1 The case must be characterised by:

- Indicative clinical and radiological data;
- Diagnosis of discharge of mesothelioma (the discharge code alone is insufficient, it is necessary to assess the medical record to check that it is not a neoplasia from another site in the body affecting the pleura). Cases in which the records have not been examined are not included in this category;
- Absence of histological examination;
- Absence of cytological examination.

3.2 The case must be characterised by:

- Death Certificate Only (DCO) with presence on the ISTAT death certificate of the term “mesothelioma”.

Cases in which the death certificate has not been examined are not included in this category;

- Absence of histological examination;
- Absence of cytological examination.
- Absence of clinical and radiological data.

4 CASES TO BEDEFINED

This category must be only used as a “temporary container” in which to classify the cases undergoing verification. Cases occurring in resident persons in another Region must be reported to the relevant COR which will then report them to the ISPESL.

4.1 The case must be characterised by:

- Histological or cytological examination enclosed in paraffin, in the absence of a characteristic morphological pattern (doubtful case), in the absence of positive diagnosting imaging for mesothelioma or mesothelioma discharge diagnosis or similar assessment made by a clinician.

or

- Immunohistochemistry not carried out, with doubtful immunophenotypical pattern, in the absence of positive diagnostic imaging for mesothelioma or mesothelioma discharge diagnosis or similar assessment made by a clinician.

4.2 The case must be characterised by:

- Hospital Discharge Forms (SDO) diagnosis only, without reading of the medical record and without further diagnostic data.

4.3 The case must be characterised by:

- Only DCO defined on the basis of the ICD IX death code, without reading of the death certificate.

5 NON MESOTHELIOMA

This is the level for cases which from level 4.0 are not able to reach a higher level (1.0; 2.0; 3.0) after having ascertained death and having waited at least two months from death (understood as the time limit for the execution of post-mortem examinations which could better define the diagnosis).

2.2 Priority in the Exposure Definition

For cases placed at level 1,2 and 3 it shall be necessary:

- to define possible exposure to asbestos. The "priority criteria" may be applied in case of reconstruction of the personal history:
  - Cases defined as "certain" placed at level 1 are to be considered "high priority" and the procedure for the exposure definition must be applied.
  - diagnostic level 2 (probable) and 3 (possible) cases shall be considered as lower priority. In these cases the procedure involving interview, determination and classification of asbestos exposure shall be performed on a representative sample provided that the remaining cases by suitably processed as soon as possible.
- to notify cases to ISPESL ISPESL (re: ReNaM).
- to record cases in the appropriate database employed by ISPESL for incidence calculations.

2.3 Criteria of definition for the incidence date

The date of the first diagnosis or incidence date places the case in the specific diagnostic level. This criteria is recommended by the European Network of Cancer Registries (*) and is applied in numerous population cancer registries.

** Taken form www.encr.com.fr Euorpean Network of Cancer Registries (ENCR) <u>Recommendations for coding Incidence Date</u>*

The date of the first event (of the six listed below) to occur chronologically should be chosen as incidence date. If an event of higher priority occurs within three months of the date initially chosen, the date of the higher priority event should take precedence.

Order of declining priority:

1. *Date of first histological or cytological confirmation of this malignancy (with the exception of histology or cytology at autopsy). This date should be, in the following order:*
    a) *date when the specimen was taken (biopsy)*
    b) *date of receipt by the pathologist*
    c) *date of the pathology report.*

2. *Date of admission to the hospital because of this malignancy.*
3. *When evaluated at an outpatient clinic only: date of first consultation at the outpatient clinic because of this malignancy.*
4. *Date of diagnosis, other than 1, 2 or 3.*
5. *Date of death, if no information is available other than the fact that the patient has died because of a malignancy.*
6. *Date of death, if the malignancy is discovered at autopsy.*

Whichever date is selected, the date of incidence should not be later than the date of the start of the treatment, or decision not to treat, or date of death.
The choice of the date of incidence does not determine the coding of the item "basis of diagnosis".

Supplementary Table 1 - Summary of the classification criteria for cases relevant to ReNaM.

	1. Malignant mesotelioma that is CERTAIN			2. PROBABLE malignant mesothelioma		3. POSSIBLE malignant mesothelioma		4. Malignant mesothelioma SUSPECTED and TO BE DEFINED		
Type of diagnosis	1.1	1.2	1.3	2.1	2.2	3.1	3.2	4.1	4.2	4.3
Histological material enclosed in paraffin	QMC Yes	QMC Yes	QMC Yes	QMD Yes	-	-	-	QMD	-	-
Cytological material enclosed in paraffin	QMC (Yes if the	QMC (Yes if the	-	QMD (Yes if the previous	-	-	-	QMD	-	-

	previous one is not available)	previous one is not available)		one is not						
Cytological material not enclosed in paraffin	-	-	-	-	QMC Yes	-	-	-	-	-
Immunohistochemistry with immunophenotypical pattern	C Yes	-	-	-	-	-	-	Not under taken or doubtful outcome	-	-
Diagnostic imaging	C Yes	C Yes	-	C Yes	C Yes	Indicative Yes	-	-	-	-
Clinical or Hospital Discharge Forms (SDO) diagnosis	C (Yes if the previous one is not available)	C (Yes if the previous one is not available)	-	C (Yes if the previous one is not available)	C (Yes if the previous one is not available)	Indicative (Yes if the previous one is not available)	-	-	Hospital Discharge Form (SDO) only	-
Autopsy diagnosis	C (Yes if the previous one is not available)	C (Yes if the previous one is not available)	-	C (Yes if the previous one is not available)	C (Yes if the previous one is not available)	-	-	-	-	-
Death certificate	-	-	-	-	-	-	Mesothelioma condition Yes	-	-	ICD IX Code only

QMC: Characteristic Morphological Pattern QMD:
Doubtful Morphological Pattern

C: Certain

YES: Necessary

QUESTIONNAIRE

3.1 –**Contents description**

Information on the subject’s work and life history with particular regard to possible asbestos exposure is collected by COR through an interview with the patient, if still alive, or with next of kin, using the standard questionnaire approved at a national level. The questionnaire is submitted by suitably trained interviewers.

The questionnaire is divided into 5 parts:

1. General Information
2. Part A – Smoking habits
3. Part B - Health
4. Part C – Professional history– general summary
5. Part D

D1 – Family background: professional history of family members or household. D2 – Environmental conditions

6. Part E – Answers Reliability
7. Part F – Professional history (industry form/trades form/other)
8. Part G – Professional history (agriculture form)
9. Part H – Armed Services history
10. Part I – Non-professional history

A specific form shall be filled out for each professional activity reported in the general summary of professional activities (Part C).

Interview type and quality data must also be reported. The revision and the critical appraisal of the questionnaire in terms of thoroughness and reliability is accomplished by Regional Operating Centres.

Additional information may be required during revision and quality assessment (e.g. subsequent direct/telephone interview; contact with co-workers and with territorial workplace health and safety services.

THE QUESTIONNAIRE

PROFESSIONAL HISTORY AND PERSONAL LIFESTYLE QUESTIONNAIRE

OPERATING CENTRE (REGION) ....

All questions refer to:........................... ....

N. of Identification................................ ....

Surname/Given Name ....

Date of Birth......../ ..../ ....Sex: ❑ M ❑ F.......................................

Place of Birth: City .... County...

Most recent residence (address). Street/road...

City. .... County..

Telephone number ....

Marital status...

If married please state year of marriage...

Educational qualification (or last school attended)..

Personal data (if the interviewed is other than the patient)

Surname /Given Name........................ ....

Date of birth........................ ....Sex:.. ❑ M ❑ F

Relative.. ....

Address...

Telephone number ....

Date of Interview......./ ..../............ ....Place of Interview ....

Interviewer..Institute ....

The interview has not been completed for the following reason:

❑ Unable to find the patient

❑ Denial of the patient

❑ Physical-emotional constraints

❑ Deceased, unable to locate next of kin.

❑ Deceased, denial by next of kin.

PART A: SMOKINGHABITS

A1.Doyousmoke?

❑ yes

❑ no

❑ have you stopped

❑ I don't know

A2. What do/did yousmoke?

❑ Cigarettes

❑ filter-tipped

❑ w/o filter

❑ both

❑ I don't know

❑ Cigars

❑ Pipe

❑ I don't know

A3.Whendidyoustartsmoking? ❑ I don't know

At what age did you stop ❑ I don't know

A4.Howmanycigarettesdo/didyousmokeperday? ❑ I don't know

PART B: HEALTH

B1. Have you had a chest X-ray?

❑ YES ❑ NO ❑ I DON'T KNOW

□ Reason .... N°...........

B2. Have you ever had X-ray therapy treatment?

❑ YES ❑ NO ❑ I DON'T KNOW

Where....................................Reason.....................Duration (months)..................

Beginning of treatment.(year)........................Therapy Location ....

B3. Do you/have you perceived an INAIL compensation for work related respiratory pathologies?

❑ YES ❑ NO ❑ I DON'T KNOW

• If so, please specify the pathology and when you perceived the compensation

....

B4. Have you ever had a major bronchopulmonary illness?

❑ YES ❑ NO ❑ I DON'T KNOW

If so, please state the pathologies…………………………………………………………………

When?………………………………………………………………..…………………………………….

B5. Have you ever had major chest traumas?

❑ YES ❑ NO ❑ I DON'T KNOW

B6. Have occurred tumour pathology cases in your family?

❑ YES ❑ NO ❑ I DON'T KNOW If

so, please state:

DEGREE OF KIN	TUMOUR	YEAR

PART C: OCCUPATIONAL HISTORY GENERAL SUMMARY

Please fill out a specific form for each work activity performed: “Trade/Industry”, “Farming”, “Military”. Report non-professional activities in the “Hobby” form.

C1. Are you working?

❑ **YES** ❑ **NO**

If not, please state reasons:………………………………………………………………

C2. Complete occupational history (including military service data).

N.	FROM		TO		NAME OF THE COMPANY	TASK
	AGE	YEAR	AGE	YEAR		

NOTE: in case of discontinuous or casual employment, state the overall duration.

Is the employment card available?

❑ **YES** ❑ **NO** ❑ **I DON’TKNOW**

C3. Do you or have you ever performed casual works?

❑ **YES** ❑ **NO**

If so, please states details in the complete professional history section.

PART D: FAMILYHISTORY

D.1 Next of kin/cohabitant working history (limited to the period of cohabitation)

Family Members (only cohabitants)

Kinship	Cohabitation (from……to…..)	Description of working activity (economic sector and tasks)	a*	Clothes**
Father				
Mother				
Spouse				
Other next of kin/cohabitant				
Other next of kin/cohabitant				
Other next of kin/cohabitant				
Other next of kin/cohabitant				
Other next of kin/cohabitant				
Other next of kin/cohabitant				
Other next of kin/cohabitant				
Other next of kin/cohabitant				

* did the job entail the use of asbestos or asbestos-containing items?

** Were workers clothing taken home?

In case of positive answer fill out the following form in column a

Relative: degree of kinship..

Surname and name ....

If deceased, state cause of death: ....

Familiarity with cancer pathology? ❑ YES ❑ NO..................... ❑ I DON'T KNOW

If positive please specify: ....

Period	Company name and location	Main products and services	Unit activity	Task and job description	Clothing (a)	Washing (b)

(If possible list only the jobs performed during the period of cohabitation)

(a) cross if workers clothing were brought home

(b) cross if workers clothing were brushed and washed at home.

Does your next of kin perceive the INAIL (Italian National Institute for Insurance against Occupational Accidents) occupational disease pension?

❑ YES ❑ NO ❑ I DON'T KNOW

If so, specify the disease and the and INAIL compensation starting date:........................

D2 Environmental condition

RESIDENTIAL HISTORY

Number of dwellings from birth to the date of diagnosis	Period (from ......to)	Municipality and county code	Address	Type of dwelling (*)

(*) Dwelling types:

1 = Rural o farm house 2 =
House or Villa
3 = Apartment
4 = Communities (boarding house, hostel, barracks, colleges, shelters)
5 = Other (Specify)

Were asbestos cement items (i.e. eternit-coverings, ceilings, water boilers, panels behind the stoves, other) present in the building after 1945?

....

....

....

Modalities and workers involved in the maintenance operations

....

The dwelling was adjacentto:

❑ Chemical/petrochemical plant............in the period ....distance.........................

❑ Thermo - electric power plant.............in the period................distance.........................

❑ Port/harbour......................................in the period................distance.........................

❑ Shipyards ....in the period................ distance.........................

❑ *Eternit* production plant......................in the period................distance.........................

❑ Railway production and repair plants..in the period................distance.........................

❑ Quarry or mine ....in the period................ distance.........................

❑ Railway line/station............................in the period................distance.........................

❑ Incinerator...in the period................distance.........................

❑ Industrial area ....in the period................distance.........................

❑ Waste disposal site ....in the period................distance.........................

❑ Other...in the period................distance.........................

Have you regularly attended public places or transports vehicles such as:

❑ Theatres..........period.................from/to.................name............. ....municipality....

❑ cinemas...........period.................from/to.................name..................municipality....

❑ gyms ....period.................from/to.................name..................municipality....

❑ pools ....period.................from/to.................name..................municipality....

❑ trains/subways/ferries period .... from/to.................name .... municipality ....

❑ other................period.................from/to.................name..................municipality....

E: COMPLETENESS OF THE DATA AND ACCURACY OF THE INFORMATION GATHERED

E1. Completeness of information

❑ Poor ❑ Partial ❑ Sufficient ❑ Good

E2. Attitude-toward the interview

❑ Positive ❑ with reserve ❑ Negative

E3. Motivation

❑ Good ❑ Sufficient ❑ Low

E4. stage of the disease

❑ Initial ❑ Advanced ❑ unapplicable (interview carried out with a substitute)

Are there other relatives or colleagues who could furnish more detailed information with respect to specific periods? If so, please indicate addresses and phone numbers.

....

....

....

Remarks:

....

....

.... Date ....

Place of the interview Interviewer signature

....

F: PROFESSIONAL HISTORY

INDUSTRY/MANUFACTURE/OTHER FORMS

(form to be used for each professional task related to the working activity)

Form related to the working activity within the company …………………………………..

From……………….to……………………address……………………………………………..

Name of the employer……………………. ....

Name of Plant manager ……………………. ....

Associated tradesmen ………………...

Colleagues ………………………………. ....

Total number of employees.............. ....

The company is: operating ❑ not operating ❑ I don't know ❑

F1.Production/finished products ....

....

....

F2. Contract type:

❑ full time

❑ part time hours per day...

❑ seasonal months per year ....

F3. General description of the working environment

....

....

F4. Detailed description of the working environment:

- Size ....
- Number of employees ....
- Machinery/equipment type and number ....

....

....

F5. Job task or activity performed ....

....

....

- Machinery and materials ....

....

....

- Machinery implementing...

....

....

- Unusual or special jobs, frequency and duration..

....

....

F6. Type of activity performed by other-workers nearby..

....

....

F7. Were dust, gases or vapours present in working place? ❑ YES ❑ NO

- If so, please specify origin ....

....

....

- Were ventilation systems fitted? ❑ YES ❑ NO
- If so, please state type?...

....

....

F8. Did you use dust personal protection equipment? ❑ YES ❑ NO

- If so, please specify type and during which period of time...

....

....

- The personal protection equipment was used to perform which task...............................

....

- Did you use heat personal protection equipment? ❑ YES ❑ NO
- If so, please specify type and during which period of time...

....

- What material was the personal protection equipment made of?....................................

- The personal protection equipment was used to perform which task? ....

....

....

F9. Were there heat emissions? ❑ YES ❑ NO

- If so, please specify ....

....

F10. Were any insulated pipes present? ❑ YES ❑ NO

If so, please specify:

- Material used ❑ I don't know
- Fluids transmitted ❑ I don't know
- Have you ever seen maintenance work involving removal of the insulation elements?

....

- Who performed the maintenance ❑ I don't know
- Were walls, ceilings and metal structures coated with insulation panels?

❑ YES ❑ NO ❑ I don't know

- If so, please specify:..

F11. Your job involved the use or direct contact with:

	Please state type:
• - Solvents	❑ ....
• - Paints	❑ ....
• - Abrasive agents	❑ ....
• - Silica	❑ ....
• - Cement	❑ ....
• - Fibrocement/Asbestos-cement/*Eternit*	❑ ....
• - Asbestite	❑ ....
• - Powder	❑ ....
• - Mineral fibres (glass/rock wool etc.)	❑ ....
• - Textile fibres or tissues	❑ ....
• - Asbestos	❑ ....
• - Other mineral fibres	❑ ....
• - Physical agents – radiation	❑ ....
• Other, please specify	❑ ....

F12. In addition to the tasks previously indicated, did you also perform maintenance work?

❑ YES ❑ NO

- If so, please specify the type of work performed (if necessary fill out an additional “industry” form)

....

....

F13. Ifmaintenanceoperationswereperformed,pleasedescribeworkcarriedout:

....

....

G: PROFESSIONALHISTORY

AGRICULTURE FORM

(form to be used for each professional task related to the working activity)

Form related to employment with company ....

From-to...............Address ....

Name of employer ....

Name of colleagues ....

Total number of employees. ....

The company is: operating ❑ not operating ❑ I don’t know ❑

G1. Description of task and activity

....

....

....

- Description of production and working environment

....

....

....

G2. Contract type:

❑ full time

❑ parttime hours per day………………………………………………………………………………

❑ seasonal months per year ……………………………………………………………………………

G3. Description of working environment: wine cellar, wineries and other beverages production facility.

• Size ....

• Number of employees...

• How would you define your work premises?

❑ Open air ❑ Storeroom/warehouse

❑ Cellar ❑ Office

❑ Tradesman workshop ❑ Laboratory

❑ Other (please specify) ....

• What kind of beverage was produced? (wine, beer, distillates, carbonated drinks, syrups, other)

....

....

• Specify your tasks ....

• Have you ever been involved in filtering operations?

❑ YES ❑ NO ❑ I don't know

• If so, please indicate average time for week:

from .... To ....

• What types of filter elements were used?

❑ Cellulose ❑ Asbestos ❑ Canvas bags ❑ I don't know

❑ Other (please specify):.................................. ....

• Was there any distillation equipment?

❑ YES ❑ NO ❑ I don't know

G4. Did you use personal protection equipment?

❑ YES ❑ NO ❑ I don't know

• If so, what was the equipment made of ....

....

G5. Were there any boilers?

❑ YES ❑ NO

If so, please specify:

• Type and location ....

• Who performed maintenance operations...

G6 Were any insulated plumbing lines present? ❑ YES ❑ NO

If so, please specify:

- Type of insulation used and what fluids did the plumbing carry.....................................

....

....

- Who performed maintenance work...

....

....

- Have you ever seen maintenance work involving removal of the insulation

elements?. ....

....

....

G7. Did the company operate a dryer? ❑ YES ❑ NO

If so, please specify: ....

....

G8. Have you ever used jute bags/sacks to store cereals, olives etc.?

❑ YES ❑ NO

If so, please specify: ....

- Were the bags/sacks new or recycled? ....
- If the bags/sacks were recycled do you recall any lettering or markings?.......................

....

G9. Have you ever used animal feedstock? ❑ YES ❑ NO

If so, specify..

....

- Type of feedstock and animal reared...
- When and how was the feedstock distributed...

G10. Have you ever used litter for stables? ❑ YES ❑ NO

If so, please specify type ....

G11. Have you ever performed repair of buildings? ❑ YES ❑ NO

- If so, please specify..
- Have you ever used asbestos-cement *(Eternit)?* ❑ YES ❑ NO
- Have you ever processed it?...

G12. Haveyoueverperformedrepairworkofmotorvehicles? ❑ YES ❑ NO

If so please specify:

- Vehicle type and work performed

G13. Have you ever used textile waste or other material as a means of relieving soil burden? ❑ YES ❑ NO

If so, please specify:

- Ageandoriginoftextilewaste...
- Other types of materials used ………………………………………………………….

H: ARMED SERVICESHISTORY

H1. Have you been in the armed Services?

❑ ENLISTMENT ❑ CAREER ❑ NO

- If so state period of time? From …………. To ………….
- During a conflict? ❑ YES ❑ NO

H2. In which force were you enlisted?...

- Where? .... From .. ..To......................................
- Please describe your duties/tasks? ....

H3. During the military service did you perform maintenance work on motor vehicles?

❑ YES ❑ NO

- If so, please specify type of vehicle and work performed: ....

....

H4.Haveyoueverdriventanksorarmouredvehicles? ❑ YES ❑ NO

- If so please specify type of vehicle: ....

....

H5. Haveyoueverbeenamemberofafire-fightingteam? ❑ YES ❑ NO

- If so please specify if you ever used asbestos or asbestos-containing items.................

....

H6. Were you assigned to gunpowder or ammunition warehouses? ❑ YES ❑ NO

- If so please state type of building and activity performed: ....

....

H7. If interviewed served in the Navy, please specify:

- Did you serve ona ship? ❑ YES ❑ NO

• If so, please state type of naval unit? ....

Year of construction .

• Please describe your tasks ....

Remark: If the interviewed was a War Prisoner, enquire if he/she performed working activities and describe them in the apposite form

I: NON-PROFESSIONAL HISTORY

I1. Did you make repairs at home? ❑ YES ❑ NO ❑ I DON'T KNOW

Specify ....

• Plumbing or electrical repairs ❑ YES ❑ NO ❑ I DON'T KNOW

Period ....

• Thermal insulation work? ❑ YES ❑ NO ❑ I DON'T KNOW

Period ....

• Masonry work ❑ YES ❑ NO ❑ I DON'T KNOW

Period ....

• Other repairs (please specify) ❑ YES ❑ NO ❑ I DON'T KNOW

Period ....

I2. Have you ever repaired your car or other motor vehicles?

❑ YES ❑ NO ❑ I DON'T KNOW

Period ....

I3. In your leisure time, have you ever performed activities that involved the use of asbestositems? ❑ YES ❑ NO ❑ I DON'T KNOW

Period ....

• Which activities?.. Period? ....

• How frequently did you carry out such activities? ❑ RARELY ❑ OFTEN

(if such activities were a second job, please furnish details in the trades/industry form) **I4. Have you ever usedtoiletrypowder?** ❑ YES ❑ NO

If so, please state: • When? .... Quantities? ....

I5. Have you ever used household items such as:

• Baking glovers ❑ period ....

- Flame protection panels ❑ period ....
- Asbestos cloth-covered ironing board ❑ period ....

• Steam iron board ❑ period ....

• Other ❑ period ....

GENERAL REMARKS

....
....
....
....
....
....
....
....
....

EXPOSURE DEFINITION PARAMETERS

Exposure shall be assessed and codified according to a classification relating to levels of certainty and modalities of exposure.

Code 1 = CERTAIN ***PROFESSIONAL EXPOSURE***

Individuals that have carried out working activities involving asbestos use/exposure. Asbestos presence must be acknowledged by at least one of the following conditions:

• explicit statement reported directly by the interviewed;

• environmental investigations, monitoring agency reports, employer's administration documents; statements reported by a relative or cohabitant;

• statements reported by relatives/other cohabitants statements concerning jobs performed in industrial sectors where asbestos use wascertified;

Individuals affected by pathological manifestations such as pleural plaques or BAL positive with professional exposure level "Probable (code2)" or "Possible (code3)";

Code 2 = ***PROBABLE PROFESSIONAL EXPOSURE***.

Individuals that worked in an industrial sector or working environment in which asbestos was certainly used or present but is not possible to document the exposure.

Code 3 = ***POSSIBLE PROFESSIONAL EXPOSURE.***

Individuals that worked in an industrial plant or in a working environment belonging to an economic sector in which the presence of/use of asbestos had been revealed but there is not information concerning individual’s use or contact (if any) with asbestos.

Code 4 = ***HOUSEHOLD EXPOSURE***.

Individuals not exposed professionally but exposed in the household due to cohabitation with at least one worker classed Code 1 or Code 2.

Code 5 = ***ENVIRONMENTAL EXPOSURE***.

Individuals not exposed professionally who lived close to industrial area using asbestos or asbestos-containing products or that may have attended for non professional reasons asbestos-containing premises.

Code 6 = ***NON-PROFESSIONAL EXPOSURE***.

The exposure is linked to activities performed in the household (use of asbestos household goods) or during leisure time (do-it-yourself, plumbing repairs, motor vehicle repairs, masonry work, etc.)

Code 7 = ***UNLIKELY EXPOSURE***.

Individuals for whom good quality data concerning professional and private history is available.

Data allows to exclude asbestos exposure levels exceeding the “natural environment background level”.

Code 8 = ***UNKNOWN EXPOSURE***.

The information collected about the individuals are incomplete and insufficient to attribute the exposure level.

Code 9 = ***UNDERGOING EXPOSURE DEFINITION***.

Additional information need to be collected in order to evaluate the exposure.

Code 10 = ***UNCLASSIFIED EXPOSURE***.

Individuals for whose cases no information is available nor will be available (closed case).
